# A Web-Based App for Emotional Management During the COVID-19 Pandemic: Platform Development and Retrospective Analysis of its Use Throughout Two Waves of the Outbreak in Spain

**DOI:** 10.2196/27402

**Published:** 2022-03-31

**Authors:** Sara Guila Fidel Kinori, Gerard Carot-Sans, Andrés Cuartero, Damià Valero-Bover, Rosa Roma Monfa, Elisabet Garcia, Pol Pérez Sust, Jordi Blanch, Jordi Piera-Jiménez, Josep Antoni Ramos-Quiroga

**Affiliations:** 1 Department of Psychiatry Hospital Universitari Vall d’Hebron Barcelona Spain; 2 CIBERSAM Universitat Autònoma de Barcelona Barcelona Spain; 3 Information Systems Directorate Servei Català de la Salut Barcelona Spain; 4 Digitalization for the Sustainability of the Healthcare System DS3 IDIBELL Barcelona Spain; 5 Sistema d'Emergències Mèdiques Sistema de Salut de Catalunya Barcelona Spain; 6 Information Systems Area Departament de Salut Barcelona Spain; 7 Faculty of Informatics, Multimedia and Telecommunications Universitat Oberta de Catalunya Barcelona Spain

**Keywords:** web-based app, emotional management, lockdown, COVID-19, posttraumatic stress disorder, anxiety, quarantine, PTSD, app, emotion, development, platform, retrospective, usage, utilization

## Abstract

**Background:**

Quarantines and nationwide lockdowns implemented for containing the spread of the COVID-19 pandemic may lead to distress and increase the frequency of anxiety and depression symptoms among the general population. During the nationwide lockdown of the first wave of the COVID-19 outbreak in Spain, we developed and launched a web-based app to promote emotional self-care in the general population and facilitate contact with health care professionals.

**Objective:**

This study aimed to describe a web-based app and analyze its utilization pattern throughout 2 successive waves of the COVID-19 outbreak in Spain.

**Methods:**

Our web-based app targeted all individuals aged 18 years or more and was designed by adapting the contents of a mobile app for adjuvant treatment of posttraumatic stress disorder (ie, the PTSD Coach app) to the general population and the pandemic or lockdown scenario. We retrospectively assessed the utilization pattern of the web-based app using data systematically retrieved from Google Analytics. Data were grouped into 3 time periods, defined using Joinpoint regression analysis of COVID-19 incidence in our area: first wave, between-wave period, and second wave.

**Results:**

The resulting web-based app, named gesioemocional.cat, maintains the navigation structure of the PTSD Coach app, with three main modules: tools for emotional self-care, a self-assessment test, and professional resources for on-demand contact. The self-assessment test combines the Patient Health Questionnaire-2 and the 7-item Generalized Anxiety Disorder scale and offers professional contact in the advent of a high level of depression and anxiety; contact is prioritized in accordance with a screening questionnaire administered at the time of obtaining individual consent to be contacted. The tools for emotional self-care can be accessed either on-demand or symptom-driven. The utilization analysis showed a high number of weekly accesses during the first wave. In this period, press releases regarding critical events of the pandemic progression and government decisions on containment measures were followed by a utilization peak, irrespective of the sense (ie, positive or negative) of the information. Positive information pieces (eg, relaxation of containment measures due to a reduction of COVID-19 cases) resulted in a sharp increase in utilization immediately after information release, followed by a successive decline in utilization. The second wave was characterized by a lower and less responsive utilization of the web-based app.

**Conclusions:**

mHealth tools may help the general population cope with stressful conditions associated with the pandemic scenario. Future studies shall investigate the effectiveness of these tools among the general population—including individuals without diagnosed mental illnesses—and strategies to reach as many people as possible.

## Introduction

In the last months, most countries worldwide have experienced uncontrolled outbreaks of the COVID-19 soon after reporting the first case. The rapid spread of its causative agent, SARS-CoV-2, has led to an unprecedented overburdening of health care systems, prompting many governments to dictate strict lockdowns for containing the outbreak.

While such indiscriminate strategies have succeeded in containing SARS-CoV-2 spread and reducing mortality [1], evidence warns on many psychological harms potentially associated with quarantines [2], particularly among older and dependent people and those with previous psychiatric pathologies [3-5]. Alongside the quarantine-specific stressors, the uncertainty associated with the pandemic scenario and impossibility of accompanying loved ones during their hospital stay or end-of-life stage has contributed to psychological overwhelming in many cases [5].

The use of mobile health (mHealth) and web-based technologies for health (eHealth) has been shown to improve access to health care services under confinement situations. In previous years, information and communication technologies (ICT) (including mHealth and eHealth solutions) for people with mental health needs have been gradually incorporated into routine care, as also for social groups not considered regular digital consumers such as older people [6]. A successful example of ICT-based solutions for mental health is the mobile app for managing posttraumatic stress disorder (PTSD; ie, the PTSD Coach app), developed by the Veterans Affairs National Centre for PTSD and the Department of Defence’s National Centre for Telehealth & Technology [7]. The PTSD Coach app has been translated to many languages―including Spanish, a translation led by our team [8]―and it is currently used in our psychiatry department as an adjuvant to face-to-face therapy for PTSD.

The Catalan Health Service, which provides universal health care to a population of 7.7 million inhabitants in Northeast Spain, has a long tradition of implementing ICT solutions for promoting integrated care and increasing the efficiency and quality of health care delivery. The overloading of the health care system experienced during the COVID-19 outbreak boosted the implementation of various digital health strategies to counteract the collapse of many health resources [9]. To our knowledge, by the time of the nationwide lockdown in our country, no mHealth solutions had been developed for supporting people with mental health needs in a quarantine or lockdown context. Thus, considering the limited access of people to the health care system and the potential of ICT-based solutions to bridge these gaps, we aimed to develop a web-based app platform for supporting emotional management among the general population during the COVID-19 outbreak in our country. The existence of the PTSD Coach app provided us with an opportunity to develop such a web-based app quickly and offer it to the local population timely.

We present herein the development, main characteristics, and launch of the web-based app, named gestioemocional.cat (“emotionalmanagement” in Catalan), and a retrospective analysis of its use throughout alternate periods of lockdown and ease of social distancing measures.

## Methods

### Design Objectives

The web-based app was developed in response to a request by the Catalan Health System and Catalan Ministry of Health to provide Catalonia citizens (Northeast Spain) with support for coping with the emotional struggle associated with the COVID-19 pandemic and the nationwide lockdown. The web-based app was designed to assess two main objectives: (1) to provide users with tools and guidance for emotional self-assessment and care, and (2) to establish a communication channel to reach health care professionals in the advent of worsening of mental health symptoms.

The web-based app targeted all individuals aged 18 years or older with the capacity to use a mobile app or website. Spain has a high smartphone penetration rate [10], for which the web-based app had the potential to reach a significant part of the population. Nonetheless, technical requirements of the web-based app were minimized to facilitate access. Furthermore, to achieve high acceptability and on-demand use of the web-based app [11], we decided to store the least information possible and not retain the user’s historical records.

### Development of the Web-Based App

The government request for developing the web-based app was made on March 26, 2020. A panel of experts consisting of 2 clinical psychologists with expertise in psychological trauma and crisis intervention and 2 psychiatrists of the Catalan Health System coordinated and oversaw the web-based app development and content generation. Considering the limited time for developing the web-based app and the effectiveness of the previously developed PTSD Coach app, we used it as a starting point to translate its contents and adapt them to a lockdown or pandemic scenario when necessary.

The panel of experts reviewed all items of the PTSD Coach app and grouped them into three categories: (1) items that could be translated into local languages without changes in their content; (2) items that required adaptation owing to cultural differences, need for broadening the clinical scope to cover anxiety and depression symptoms, or adequateness to the quarantine or pandemic scenario; and (3) items to be removed for definite inappropriateness in accordance with the intended use of the App. Owing to the urgency of the development of the web-based app, item review was based on the clinical criteria and the expertise of panel members. Of note, 2 of them had contributed to developing and translating the PTSD Coach App and had, therefore, in-depth knowledge of the rationale behind each item. The resulting contents were used to develop the web-based app using the corporate colors and design of the Catalan Health System. The authors of the PSTD Coach app kindly provided permission to adapt and translate their App.

### Time Frame and Launching Strategy of the Web-Based App

The first case of COVID-19 in Spain was officially reported in late January 2020. The Spanish government halted public gatherings on March 9, 2020, and led to a state of alarm and a nationwide lockdown on March 13, 2020. The lockdown started a progressive easing on May 10, 2020, and was definitely lifted on June 20, 2020.

The web-based app gestioemocional.cat was launched by the Catalan Health Department on April 15, 2020. Information regarding the web-based app was disseminated using television and radio advertisements, which were released through the channels of the public corporation.

### Utilization Analysis and Statistics

Data for utilization analysis of the web-based app were gathered from Google Analytics. The main objective of the utilization analysis was to describe the number of users throughout the study period. We used a script to systematize queries for retrieving information on daily accesses to the web-based app. For longitudinal analysis of the use of the web-based app, the number of events was transformed into a logarithmic scale and plotted against a timeline covering the period from the web-based app launch on April 15, 2020, to data set closure on December 16, 2020. Key events regarding outbreak progression (ie, relevant press releases on epidemiological information and government decisions) and the time window of each wave of the outbreak were added to the plot. The 2 waves of the COVID-19 outbreak were defined using Joinpoint regression analysis of publicly available data on COVID-19 incidence in our area. A permutation test with 0.05 confidence and 4499 permutations was used as described elsewhere [12]. Differences in the distribution of test scores on each of the priority levels in the 3 study periods (ie, first wave, second wave, and between waves) were assessed using a Pearson chi-square test and setting the significance threshold at a 2-sided α value of .05. The rest of the analyses were descriptive, and no other hypothesis tests were conducted. All analyses and plots were performed using R package [13].

## Results

### Platform Components

**Figure 1:**
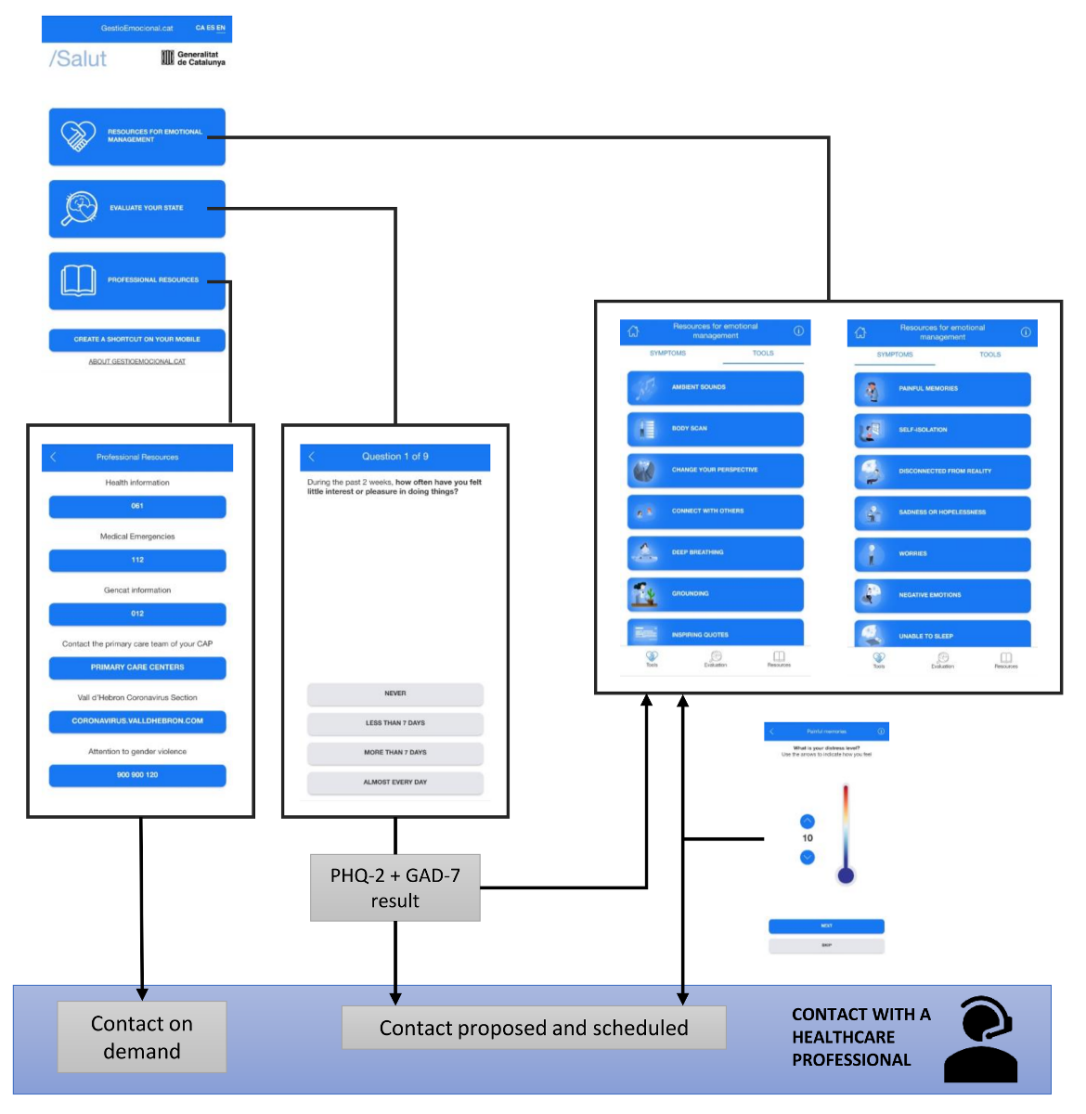
Navigation structure of the gestioemocional.cat web-based app. CAP: primary care center (for the Catalan acronym "Centre d'Atenció Primària"), GAD-7: 7-item Generalized Anxiety Disorder scale, PHQ-2: Patient Health Questionnaire-2.

The gestioemocional.cat web-based app has the same navigation structure as the PTSD Coach app. Table 1 summarizes the changes introduced to the PTSD Coach items to adapt them to the intended use of the gestioemocional.cat web-based app; the resulting list of items was translated to the local languages (ie, Spanish and Catalan). Figure 1 outlines the navigation structure of the web-based app. The main menu provides the user with access to three sections: (1) resources for emotional management, (2) tools for self-assessment, and (3) professional resources.

**Table 1:** Summary of content changes for adaptation of the PTSD Coach app into gestioemociona.cat web-based app^a^.

PTSD Coach app contents		Gestioemocional.cat contents	Rationale for change
**Main menu**			
	Manage symptoms	Resources for emotional management	Wording change (no specific syndrome)
	Take assessment	Self-assessment tools	—^b^
	Health care resources	Health care resources	—
	Learn	—	Intended for an on-demand use; no personal history recorded
**Symptoms**			
	Remind of trauma	Painful memories	Wording adapted to a broader scope
	Disconnected from people	Self-isolation	Wording adapted to a quarantine context
	Disconnected from reality	Disconnected from reality	—
	Sad or hopeless	Sadness or hopelessness	—
	Worried or anxious	Worries	Wording adapted to a broader scope
	Angry	Negative emotions	Wording adapted to a broader scope
	Unable to sleep	Unable to sleep	—
**Tools**			
	Ambient sounds	Ambient sounds	—
	Body scan	Body scan	—
	Change your perspective	Change your perspective	—
	Contact with others	Contact with others	—
	Deep breathing	Deep breathing	—
	Grounding	Grounding	—
	Inspiring quotes	Inspiring quotes	—
	Leisure activities	Leisure: time alone	Wording adapted to a quarantine context
	Mindfulness	Mindfulness	—
	Muscle relaxation	Muscle relaxation	—
	Observe thoughts	Observe thoughts	—
	Relationship tools	Relationship tools	—
	Sleep tools	Help falling asleep	—
	Soothe the senses	Soothe the senses	—
	Soothing images	Soothing images	—
	Thought shifting	Thought shifting	—
	Time out	Time out	—
	RID: coping with triggers	(Removed)	In a non–posttraumatic stress disorder setting, no specific trigger is expected
	Calming thoughts (user uploaded)	(Removed)	No personal section or user history was included
	Schedule worry time	(Removed)	Intended for an on-demand use
	Positive imagery	(Removed)	No personal section or user history was included
	My feelings	(Removed)	No personal section or user history was included
**Self-assessment**			
	3 items (evaluate your state, assessment history, and schedule self-assessment)	Evaluate your state	No personal section or user history were included
	PTSD Checklist for DSM-5^c^	Patient Health Questionnaire-2	Broadening of assessment scope (anxiety and depressive symptoms expected)
		Generalized Anxiety Disorder-7	Broadening of assessment scope (anxiety and depressive symptoms expected)
**Professional resources**			
	User can choose or be redirected toward crisis resources, professional care, or choose persons in his/her social network	Exhaustive list of telephone numbers for general health and mental health support	Adapted to local resources

^a^Unchanged contents (ie, rationale for change dashed) were directly translated to the local languages (ie, Catalan and Spanish) without content changes.

^b^—: not available.

^c^DSM-5: Diagnostic and Statistical Manual of Mental Disorders, Fifth Edition.

#### Resources for Emotional Management

The section “resources for the emotional management” can be accessed through two pathways: symptom-guided and resource type–guided (Figure 1). Symptoms include painful memories, self-isolation, disconnection from reality, sadness or hopelessness, worries, negative emotion, and sleeping disturbances. All these symptoms have been identified as very common during quarantines and lockdowns in the context of the COVID-19 pandemic [14,15]. Resource types include body scan (ie, audio-guided), change one’s perspective (ie, random text messages to rationalize distress thoughts), connect with others (ie, list of ideas or tips for establishing interpersonal relationships under a quarantine context), deep breathing (ie, audio-guided technique for emotional regulation trough breathing), grounding (ie, list of tips for helping to be fully present for whatever is occurring right here and now), inspirational phrases, use of personal leisure time (ie, list of proposed activities to be performed during the lockdown), mindfulness exercises, progressive relaxation, observe thoughts (ie, audio-guided techniques to help coping with distress thoughts), relationships improvements (ie, list of tips to improve peer communication), help falling asleep (ie, list of proposals for sleeping better), calm the senses, relaxing images, thought shifting (ie, list of phrases to be repeated over 5 minutes using a countdown tool), and time-out (ie, list of proposals to change the environment when remaining quarantined).

Irrespective of the pathway chosen to access the section “resources for the emotional management,” the web-based app suggests a particular resource based on the user’s distress level, assessed using a single-item questionnaire (ie, what is your distress level? [use the arrows to indicate how you feel]) and rated on a 10-point scale with scores ranging 0-10. Users can select the distress level either stepwise―using up and down arrows―or using a distress thermometer with an intensity color code (Figure 1).

#### Self-assessment

The self-assessment section is based on two questionnaires for a mental health assessment with a Spanish version adapted and validated: the Patient Health Questionnaire-2 (PHQ-2) [16] and the 7-item Generalized Anxiety Disorder scale (GAD-7) [17]. The PHQ-2 is intended as a first-step approach to identify depressive symptoms on the basis of the frequency of depressed mood and anhedonia. While not recommended for depression diagnosis or monitoring, it is considered useful for screening for depression, which is the most common psychological conditions in clinical practice and research; individuals who screen positive should be further evaluated with the PHQ-9 to determine whether they meet the criteria for a depressive disorder [18]. The Spanish version of the PHQ-2 has shown good reliability and validity in identifying depressive symptoms in the primary care setting [19]. The GAD-7 was developed for its use in primary care, and it has high sensitivity in identifying symptoms of the anxiety spectrum. The Spanish validation of the questionnaire has shown good reliability and validity [20]. To facilitate self-assessment, the 2 questionnaires were merged into a single questionnaire. The combination of the PHQ-2 and GAD-7 for a joint assessment of anxiety and depression has shown high internal reliability and strong convergent and construct validity when assessing its association with other mental health, quality of life, and disability measures [21]. All items of the combined questionnaire refer to the frequency of symptoms within the past 2 weeks and are rated on a 4-point scale where 0=“never,” 1=“less than seven days,” 2=“more than seven days,” and 3=“almost every day.”

**Figure 2:**
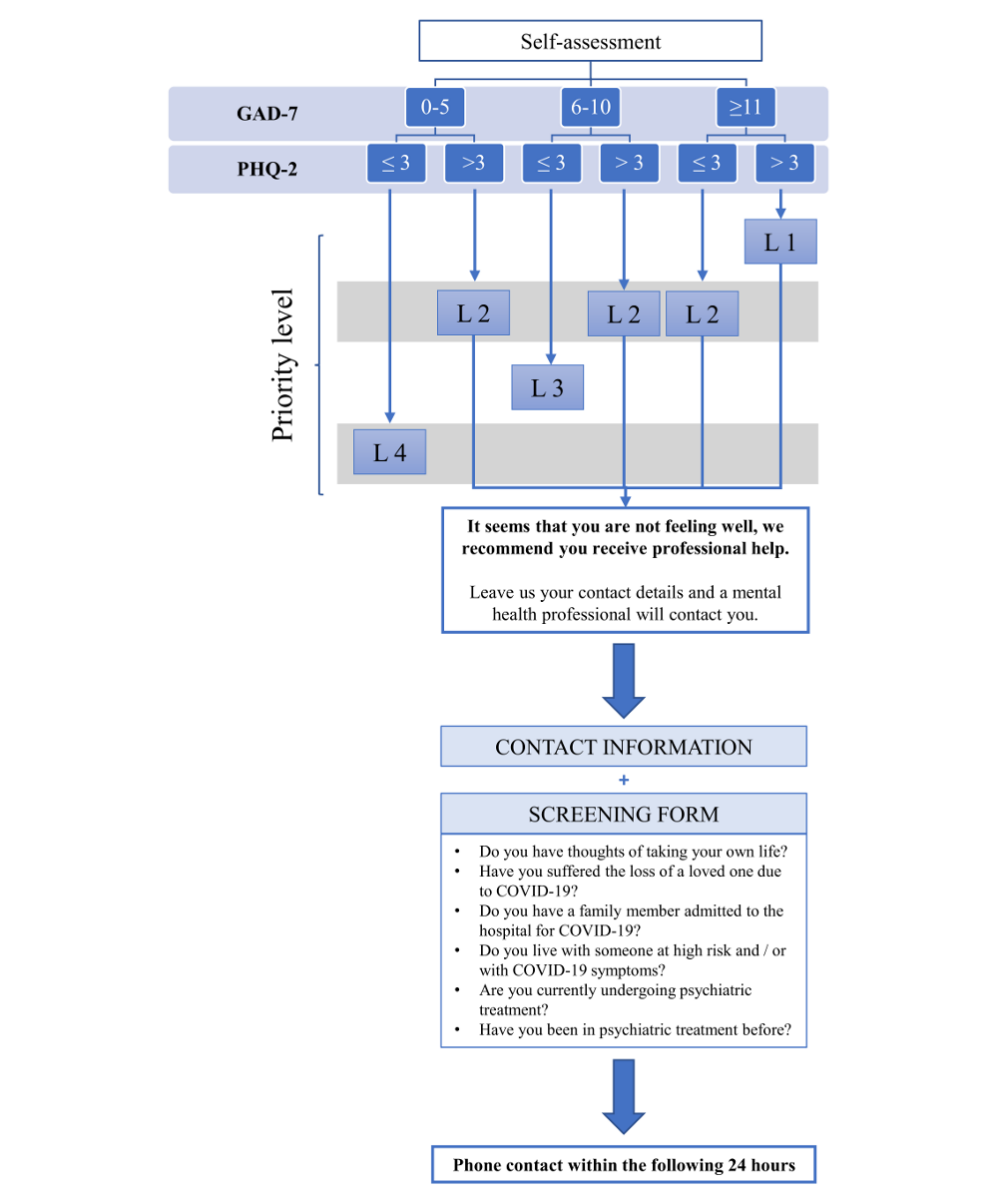
Prioritization algorithm for proactive contact with health care professionals on the basis of the assessment of mental health status. GAD-7: 7-item Generalized Anxiety Disorder scale, PHQ-2: Patient Health Questionnaire-2.

The results of the 2 questionnaires are combined to provide 4 priority levels of care needs (Figure 2). Users with low scores in the PHQ-2 and GAD-7 (ie, suggestive of mild symptoms of anxiety and depression) are encouraged to the routine use of self-management and self-assessment tools to either maintain (level 4) or improve (level 3) their emotional health status. Conversely, users with high scores on the PHQ-2 and GAD-7 (ie, suggestive of severe anxiety and depressive symptoms) are offered telephone contact with professionals of the emergency medical service. Users who agree to be contacted by a health care professional are redirected to a consent form, which collects minimum personal data. Contacts are prioritized on the basis of the combined result of the self-assessment questionnaire (ie, 1 or 2) and a screening form asking about 6 extreme mental health conditions: suicidal ideation, loss of a loved one due to COVID-19, family member admitted to hospital owing to COVID-19, living with someone at high risk of severe illness or with COVID-19 symptoms, current psychiatric treatment, and previous psychiatric treatment. Affirmative responses to these questions were assigned the highest priority, irrespective of the self-assessment questionnaire results (Figure 2).

Scheduled telephone calls are performed by the same professionals attending calls from people who reach the emergency medical service seeking mental health support. Once the phone contact has been established, health care professionals perform an independent assessment, irrespective of the contact source (ie, direct call to emergency medical services or scheduled call through the web-based app).

#### Healthcare Resources

The section “healthcare resources” allows the user to proactively contact community resources, including general health information, emergency medical services, general government information office, the user’s primary care center, and the office for women under gender violence. Additional resources include the Galatea Foundation, aimed at providing support to health care professionals, the Official College of Psychologists, which can respond to population demands and transfer cases to public health care professionals, and the Vall d’Hebron Coronavirus Section, a web-based platform available 24 hours a day, 7 days a week for attending concerns related to emotional and social impact of the COVID-19 pandemic.

### Utilization

**Figure 3:**
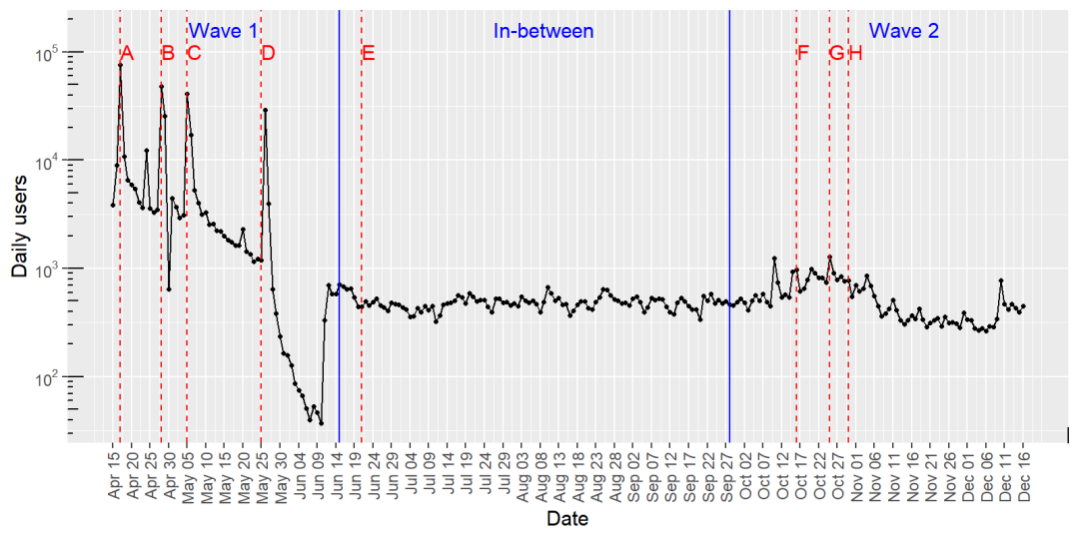
Longitudinal analysis of the number of accesses to the web-based app (logarithmic scale). Each time point shows the cumulative number of users in the 5 previous days. Red dotted lines show press releases of key events and government decisions regarding the COVID-19 outbreak. A: April 17, 2020—the Catalan Ministry of Health reports and posts the highest RT-PCR–positive and hospitalization rates. B: April 28, 2020—the Spanish government announces a de-escalation plan, to be deployed in 4 phases. Catalonia exceeds 10,000 deaths due to COVID-19. C: May 5, 2020—the government announces an upcoming partial lockdown. D: May 25, 2020, Barcelona—the greatest area in Catalonia, enters de-escalation phase 1. E: June 21, 2020—the whole country officially starts the “new normal” state. F: October 16, 2020—the government dictates a temporal lockdown of bars and restaurants. G: October 25, 2020—the government dictates nationwide curfew from 10 PM to 6 AM. H: October 30, 2020—the government dictates a permanent closure of area boundaries and weekend closure of city boundaries. The time window of each period (ie, first wave, between waves, and second wave) was defined using a Joinpoint regression analysis. RT-PCR: reverse transcription–polymerase chain reaction.

Between April 15, 2020 (ie, the launch date), and December 16, 2020, the web-based app reported 582,826 accesses. Figure 3 shows the longitudinal analysis of the number of users and critical events of pandemic progress. During the final period of the nationwide lockdown, the daily number of users peaked concomitantly with key news or government actions released by the press. Most of the press releases announcing changes on containment measures were immediately followed by a utilization peak, irrespective of the sense of these changes (ie, increasing or easing restrictions). Immediately after announcing the easing of restrictions (eg, the start of the de-escalation plan), the daily number of users remarkably decreased. The decrease was particularly abrupt when Barcelona, the most populated area, entered the first phase of the de-escalation plan.

The number of users during the second wave of the outbreak was persistently lower than that in the first wave. During this period, use of the web-based app was less sensitive to the announcement of government measures.

### Progression of Self-assessment

**Figure 4:**
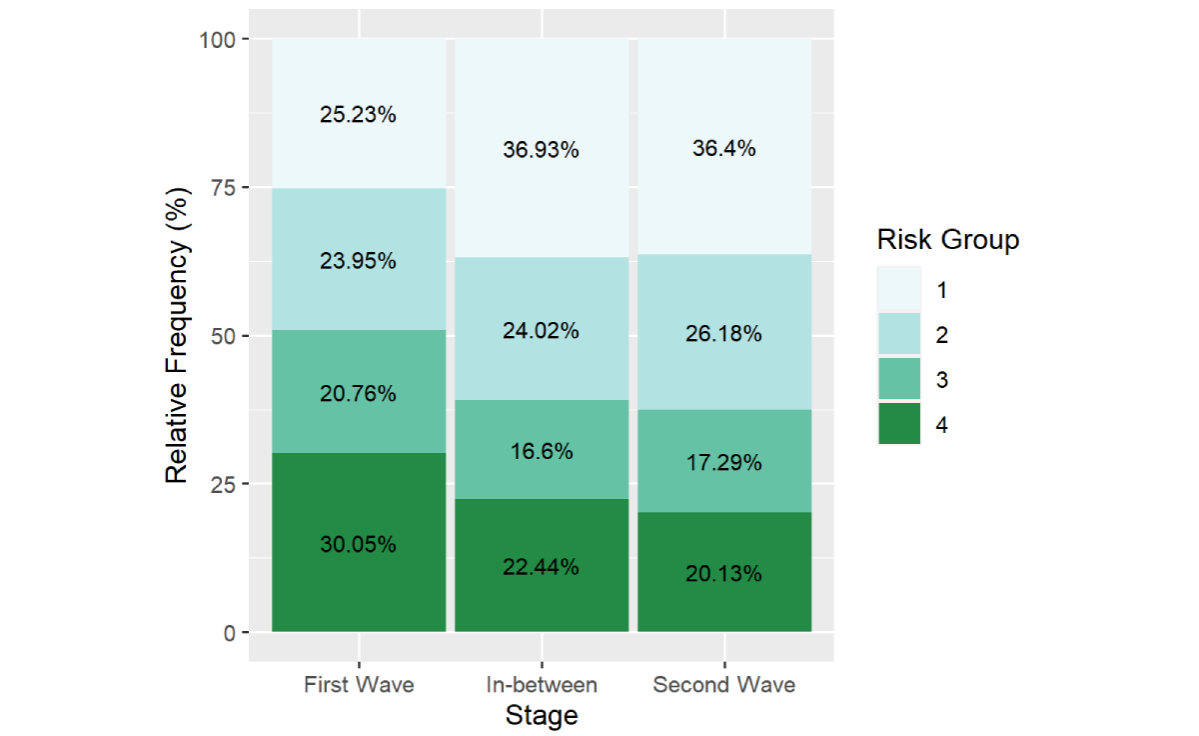
Distribution of the results of the self-assessment test across priority levels (ie, risk group) in each of the analysis periods: first wave (n=124,087), between waves (n=8510), and second wave (n=9740). The 3 periods were defined using a Joinpoint regression analysis. The Pearson chi-square test for percentage distribution was significant at *P*<.001 (*χ*^2^_6_=1349.1).

During the study period, the web-based app recorded 142,337 self-assessment tests: 124,087 (87.2%) during the first wave, 8510 (6.0%) during the period between waves, and 9740 (6.8%) during the second wave. The chi-square test revealed significant differences in the distribution of percentages across the priority levels (obtained from the self-assessment module) between the analysis periods (Figure 4). The percentage of tests scoring for priority levels 1 or 2 (associated with more severe symptoms of anxiety and depression) was higher in the second wave and between-wave periods. Overall, the offer of professional help was accepted in 8588 cases (11.4% of all cases with priority levels 1 or 2 and, therefore, triggering the message for an offer of professional help).

## Discussion

### Principal Findings

In this study, we describe a web-based app aimed at providing the general population with self-care and self-assessment tools for emotional health during the lockdown associated with the COVID-19 pandemic and establishing a new communication channel between the population and health care professionals. In addition, to reporting the characteristics of the web-based app, we analyzed the utilization pattern of the web-based app during the first 10 months of the COVID-19 outbreak in Spain. The frequency of use of the web-based app was particularly high during the first wave of the outbreak in Spain and progressively declined until reaching a baseline activity below 1000 weekly accesses after halting the national lockdown. During the first wave, the number of accesses peaked after press releases associated with the pandemic progression or changes in the containment measures dictated by the government. Interestingly, positive news (eg, relaxation of social distancing measures) were immediately followed by a sharp increase in utilization and a successive decline. The between-wave and second wave periods were characterized by a less responsive pattern of web-based app users.

The self-assessment module of the web-based app, designed to work as a sentinel for detecting severe mental health symptoms and proactively offer direct contact with health care professionals, was mostly used during the first wave. However, the scores of the self-assessment questionnaire were higher after the first wave, suggesting a higher proportion of people with mental health care during the second wave and between-wave periods.

### Limitations

Development and launching of the web-based app were strongly influenced by the urgency and overloading of the health care system, which challenged the design of an adequate assessment strategy of the platform. We chose the PTSD Coach app because of the symptom overlapping between PTSD and those reported elsewhere for lockdown situations, particularly regarding anxiety and depression. One of the advantages of the PTSD Coach app was the body of evidence on its effectiveness for self-management of mental health needs. However, owing to the shift in the target population (ie, from people diagnosed with PTSD to the general population), effectiveness of our web-based app cannot be assumed and should be confirmed in future studies. Of note, the complicated scenario in which the web-based app should be assessed will strongly challenge the trial conduct.

Another important limitation of our retrospective analysis was the limited data stored, which precluded more exhaustive assessments of behavior patterns among users (eg, to discriminate between entries of new users and regular access of the same user). However, because of the negative correlation that often exists between App use or demand and permissions for accessing private data [11], we were concerned that collecting too much data from users might be a barrier to web-based app utilization. Of note, the government announcement of launching a COVID-19 tracer app raised many privacy concerns among the population and was strongly criticized. In light of this general perception, we prioritized the acceptability of the web-based app over the capacity of collecting personal information. As a result, our database for the retrospective analysis lacked important information that might have helped to understand behavior patterns among users. One of the most remarkable consequences of this limitation is the lack of data at a user level, which precludes discriminating between entries of new users and regular access of the same user. More importantly, the lack of historical records of users prevents us from analyzing improvement or worsening of mental health state. Finally, we could not analyze the usage of the web-based app in accordance with socioeconomic status, which has been highlighted as a source of inequality in other aspects associated with access to COVID-19 resources [22,23]. Nevertheless, while all these limitations compromise analytical approaches of the web-based app, the absence of personal questions and no need for signing in are likely to promote the platform use, in line with the overarching goal of the project, which was to help the population during the stressful circumstances of the lockdown.

### Comparison With Prior Work

To our knowledge, by the time of developing the web-based app, no other mHealth solutions for promoting emotional self-care in the general population during a lockdown or quarantine situation had been launched or published. The closest example of an mHealth tool that matched our aim was the PTSD Coach app, which was innovative in the sense that users can access tools through two alternative pathways: on-demand and symptom-driven [7]. Although the PTSD Coach app was designed as adjuvant to face-to-face therapy for PTSD, its authors suggested that it could also help people not receiving psychological treatment [24,25]. Further experiences also showed good usability and ease of cultural adaptation of the App [8], encouraging us to adapt its contents to a lockdown and pandemic scenario in our country.

Although we cannot directly compare the utilization patterns of our web-based app with other mHealth or eHealth solutions for lockdown contexts, the trends observed in our analysis are consistent with information reported elsewhere. For instance, the increased consumption of alcohol and drug abuse reported during this period in our area [15] could likely be attributed to increased anxiety symptoms, grief processes, and difficulties associated with intensive cohabitation [26,27]. Similarly, other countries have reported increased demand for attention from people with mental health needs during nationwide lockdowns associated with the COVID-19 pandemic [28,29].

The design of our retrospective analysis precludes drawing strong conclusions regarding the reasons underlying the observed trend toward a less responsive usage of the web-based app during the second wave of the outbreak. However, this finding is consistent with the “pandemic fatigue” phenomenon highlighted by the World Health Organization and defined as an expected and natural response to a prolonged public health crisis [30]. This type of reaction might correspond to M Seligman’s Paradigm on learned helplessness, which describes a passive behavior exhibited by a subject after enduring repeated aversive stimuli beyond their control. It is worth noticing, however, our finding that self-assessment tests more often scored priority levels 1 or 2 during the between-wave and second wave periods suggests that people with higher mental health needs were more likely to retain the usage of the web-based app after the first wave.

Finally, the fact that 8588 self-assessment tests ended up with a scheduled telephone call with health care professionals for mental health support indicates that the web-based app has emerged as an additional communication channel for seeking professional help. The number of telephone contacts represents a low percentage (11.4%) of the total situations in which professional support was offered. However, this finding is consistent with current evidence, which suggests that most people with common mental health problems do not seek professional help [31,32].

### Conclusions

In the first year of the COVID-19 pandemic, various authors have emphasized the need to promote original and creative ways to help people cope with the new and stressful conditions during the pandemic [33,34]. We did not want to miss the opportunity to adapt mHealth tools with proven efficacy in self-management of stressful situations to the pandemic scenario. Future studies shall investigate the extent to which this tool helps people cope with the pandemic scenario and address strategies for making as many people as possible aware of this resource.

## Abbreviations

GAD-7: 7-item Generalized Anxiety Disorder scale
ICT: information and communication technologies
mHealth: mobile health
PHQ-2: Patient Health Questionnaire-2
PTSD: posttraumatic stress disorder
